# Case of Twisted Pedicle

**Published:** 1877-12

**Authors:** K. Stansbury Sutton

**Affiliations:** Pittsburgh, Pa.


### CASE OF TWISTED PEDICLE. OVARIOTOMY.

By R. Stansbury Sutton, A.M.,M.D., Pittsburgh, Pa.

On the morning of August 14, 1877, I was requested by Mr. B. to see his wife. He said, "She is very large and has dropsy." Proceeding to the house with my aspirator, I found a lady, aged 31 years, lying in bed on her right side, and in great distress. Her respirations were short, purely pectoral and 40 to the minute. Her pulse was 136, tongue red and dry. She was pale, and the expression of her face anxious. Her legs were not œdematous. It was impossible for her to lie either upon the back or left side. Her abdomen was immense; above a line drawn obliquely from the middle of the left lumbar to the centre of the right hypochondriac region, she was tympanitic; below this line dullness in percussion was general and fluctuation distinct. She was not tender to firm pressure made at all points. From the husband I learned that she had been very large for at least two years, and that he had married her one year before, not suspecting any special cause for her unusually full abdomen; that she had become pregnant, and that just ten days before, she had miscarried a six months fœtus weighing about two lbs. From the nurse, an intelligent woman of ample experience, I learned that, on the fourth day after this, she had complained of thirst and become feverish. No evidence of metritis or pelvic cellulitis was present, and I concluded that I had to deal with an ovarian cyst, the pedicle of which had become twisted, and that commencing gangrene of the cyst was setting up peritonitis. I gave this opinion to the husband with a grave prognosis; in the event of leaving her alone that death would follow in a very few hours; in the event of resorting to treatment that but little hope of saving her life could be entertained, but that such a course gave the only chance of hope. The young wife begged for the latter course, and the husband and father of the patient assented. To gain time, and to relieve the extreme urgency of the symptoms, I thrust into the cyst an aspirating needle, and drew off twelve pints of a dark chocolate-colored fluid, which I found rich in albumen, containing the compound granular cell, all of which confirmed my diagnosis of the character of the cyst. I ordered her stimulants and animal broth with citrate of lithia, and left the house. Returning to my office I gathered my instruments together, and taking with me Drs. McCann and Lemoyne, I returned to my patient at 4 P. M. We found her respiration improved, 24 to the minute, her pulse 132. Her measurements were as follows: Around the abdomen at umbilicus, 46 inches; two inches below, 46 inches; from umbilicus to right supra-spinous process of ilium, 12½ inches; from the same to the left, 12½ inches; from ensiform cartilage to umbilicus, 9½ inches; from umbilicus to pubis, 10½ inches. My friends both examined her carefully, and coincided with me in the diagnosis, and in the opinion given as to treatment. Her husband had gone to the city on some urgent business, and would not return until too late to operate by daylight. Not feeling satisfied with his absence, and being certain that she was getting into better condition as to respiration and circulation, we agreed to postpone operating until 8 A. M. of the next morning.

In addition to what she was taking, we gave her small doses of morphia during the night.

Aug. 15, 8 A. M. Present, Drs. McCann and Lemoyne. Pulse 132. Temperature 100½. Has slept about three hours during the night, and says she feels better than she did yesterday. Assisted by Drs. McC. and L., I performed ovariotomy, removing 27 pints of fluid and a cyst with a large solid portion, globular in form, weighing three and a half lbs.; making with the fluid drawn yesterday 39 pints (weighing 39 lbs.) all added to the weight of the cyst and solid contents, 42½ lbs.

The adhesions were all recent, and omental general peritonitis was present, the omentum being very red, and at least four inches square of its surface peeled off from the cyst as it was drawn out. On the right side, several patches of peritoneum were congested and red. The abdominal wound was about five inches in length, and was made with carbolized instruments; no ligatures were required, and during the entire operation not over an ounce of blood was lost to the patient. The pedicle was twisted one-half revolution only. It was broad, of medium length, and was to the left of the uterus. It was tied with carbolized silk, the knots cut short, and the cyst was cut away an inch above, and just below the solid growth alluded to, in the bottom of the cyst. The abdomen was carefully cleaned out with warm carbolized water, and carefully prepared sponges of the finest texture. The wound was closed with silver wire sutures, including the peritoneum. A few straps of adhesive plaster, some layers of carbolized lint, a thin layer of cotton wool and a broad bandage completed the dressing. Some brandy was now given, and the patient put to bed with a jug of hot water to her feet, and a blanket over her. She slept for two hours. At 1:30 P. M. her pulse was 132. Temperature 99⅗. Has had no nausea, and taken a few spoonsful of milk and lime water and some brandy. At 3 P. M.

took some citrate of lithia in solution, also a little milk. At 4 P. M. took some brandy, and passed water voluntarily. At 5 P. M. pulse 144. Temperature $99\frac{7}{8}$. An injection, consisting of five grains of quinine, ten drops of tincture of opium, and two ounces of milk, to be thrown into the rectum every four hours. A tablespoonful of iced milk to be given by the mouth as often as the patient desires liquid. A tablespoonful of brandy to be given by the mouth every four hours. No pain is complained of, but the patient expresses herself as comfortable.

8 A. M. Aug. 16. Twenty-four hours since we met to operate yesterday morning. The nurse reports that the patient was very quiet all night, and frequently passed gas from the bowel. She is yet very tympanitic, pulse 140, temperature $101\frac{3}{4}$. Has taken during the night considerable milk. Requests beef tea this morning. To the rectal injections I ordered added a tablespoonful of whisky every two hours. The gas to be drawn from the rectum with a large male catheter each time before the injection is given. By the mouth, to take a tablespoonful of whisky and two ounces of beef broth every two hours.

4 P. M. Dr. Lemoyne present by my invitation. Pulse 135, temperature $101\frac{4}{5}$. Expression of countenance better than in the morning; tongue dry. Has no pain. No pouching of Douglas' cul-de-sac. Kidneys acting well, urine clear. No catheter required. Treatment continued.

10 P. M. Pulse 136, temperature $100\frac{3}{4}$. Aug. 17, 9 A. M. Pulse 160, respiration rapid, temperature 95. 10:12 A. M. Died without a struggle.

The nurse reports that she slept from 10 P. M. until a few minutes of 1 o'clock A. M. After waking, took some beef broth and brandy, but was restless until 3 A. M., when she observed her sinking rapidly. Thirty minutes before she died she took with her own hand and drank a glass of whisky and water. Death occurred from shock. No autopsy was granted.

The following fact in the microscopical character of the fluid is important. It contained, in addition to the elements mentioned, a very large proportion of blood corpuscles. Hemorrhage into the cyst had been considerable. The arteries of the rotated pedicle still let in the blood, which the veins were incapable of returning.

The appearance of the cyst, when viewed through the abdominal incision, was as follows: Its color was changed to a dark red hue, showing active inflammation over a large surface; to this the omentum was attached by recent adhesions; along the border of this inflamed surface gangrenous lines were well defined. After removal, an examination of the cyst proved that it was rapidly becoming gangrenous throughout. The large solid mass in the bottom of the cyst was laid open, at its equator, with a knife, and was found to be full of cavities, from the size of a filbert to the size of a walnut; all of these cavities were filled with pus. The weight of the cyst and solid lump was 3½ lbs. The weight of the fluid 39 lbs, making 42½ lbs.

It is my opinion that had the condition been recognized three or four days earlier, the operation would have been more likely to have saved her. Or had ovariotomy been performed during pregnancy, still a better prospect would have been afforded her.

